# The Ganglionic Nervous System; Its Structure, Functions, and Diseases

**Published:** 1859-05

**Authors:** 


ART. III.—*The Ganglionic Nervous System; its Structure, Functions and Diseases.* By JAMES GEORGE DAVEY, M.D., M.R.C.S., London Physician (now) to the Stapleton General Dispensary, near Bristol, formerly Medical Superintendent (F.D.) of the County Asylums for the Insane at Hanwell and Colneyhatch, Middlesex. London: John Churchill, 1858. 1 vol. 8vo., pp. 309.

THE broad title with which Dr. Davey prefaces his work, must not be regarded as an accurate index of its contents. The book is not a systematic treatise on the ganglionic nervous system, but an exposition of certain views concerning its office and functions. The author himself, after rehearsing his subject in a long introduction of thirty-six pages, seems to have become conscious of some discrepancy between the caption of his proposition, as set forth in the title-page, and its demonstration, and tells us, in an apologetic way, at the commencement of the chapter on the structure of the sympathetic, "that his book is essentially physiological; but that he has thought it necessary to the entirety of the subject and the easier comprehension of the views therein unfolded, to prefix some general remarks on the anatomy of the sympathetic nerve." This statement reveals the secondary character of the anatomical portion of the work, and drives us elsewhere in search of the author's object in publication. By gleaning, here and there, a little from many obscure pages, we find, underlying the whole volume, the hypothesis that the ganglionic nervous system is the seat and origin of all vital phenomena. The mode by which Dr. Davey arrives at this startling conclusion is thus: he first assumes the main point at issue, and asserts that vitality is not an inherent property of organized tissues, but is always associated with and dependent upon nerve structure; this taken for granted, he proves that vital phenomena may exist and be manifest when the cerebro-spinal nervous system is absent, and concludes, therefore, that they must originate in the remaining nerve structure, that of the sympathetic. It is scarcely necessary to say more than this, to show the illogical character of the book. However, let us examine more in detail, the author's treatment of his subject.

He portions his work into four chapters, one to each of the following headings: The Structure, Functions, and Pathology of the Ganglionic Nervous System, and general observations on the Treatment of Disease. The object of the anatomical chapter is to show the magnitude and universal distribution of the sympathetic system, and that the brain and spinal cord are subordinate to it. These points are necessary to illustrate the capability of the ganglionic system to be the seat of life, and the little probability that such an important function would reside in a subordinate organ. The author says:—

"When, in the course of these pages, I shall have to treat of the many and important functions of the ganglionic nervous system, it may strike the mind of the reader that its mere bulk is hardly compatible with the offices claimed for it in the animal economy; however, this objection as regards the mere quantity of nerve tissue is rather apparent than real." "So universally and copiously is the ganglionic nervous tissue distributed, that, like the parenchymatous tissue, it is interwoven with every portion of the organism; and more even than this, constitutes a great part of the volume and weight of the whole body."

To establish the presence of ganglionic nerve matter in cellular animals, etc., where it escapes detection by the closest scrutiny, we have the ingenious supposition of a "highly elaborated (imperceptible?) nervous organism." The author offers no proof of such a condition, but thus announces his belief:—

"I cannot myself doubt that nervous matter *is* present in a 'diffused form,' *i.e.* incorporated with the other tissues, in all those animals wherein no *distinct* nerve structures have been made out. I cannot dissociate the positive *vitality* of such animals from a highly elaborated (imperceptible?) nervous organism."

Touching the last point, the supremacy of the ganglionic system, the author rests his faith, mainly, on his belief of its precedence of development both in the animal series and in those individuals possessed of two nervous systems—sympathetic and cerebro-spinal. He writes—

"It is concluded, then, the ganglionic nervous system first exists in a *molecular* form; and that it is made up of *globules* dispersed throughout the *homogeneous* texture of the animal, as in the acriti, the lower entozon, etc.; as such, it has always been presumed to exist in the early human embryo."

"These nervous globules, arranging themselves in a longitudinal series, form filaments, and these "filaments" at length form a central point, a ganglion (*solar ganglion*, Anderson.)

"Ascending the scale of animal creation, and arriving at the mollusca, nervous matter is found secreted about and around the œsophagus, and on the dorsal aspect of the animal (supra-œsophageal ganglion;) this nervous matter becomes, in the higher animals and in man, the tubercula quadrigemina or medullary oblongata.

"Still ascending, the cyclo-ganglionic nervous system becomes more complicated, while the several tissues and structures subordinate to the same, including the cerebro-spinal system, are proportionately amplified; the increased development of the former, and the general augmentation and addition of parts in the latter, being in the relation to each other as *cause* and *effect*."

Such, then, is the anatomical chapter; how far it illustrates the structure of the sympathetic each can judge. In the physiological section, which is the one that professedly characterizes the work, an attempt is made to demonstrate that such phenomena as irritability, formative power, animal heat, instinct, etc., are functions of the sympathetic.

Early in the introduction we have brought to our notice the author's peculiar opinion concerning the source of irritability. He presents it as an extension of, or corollary to the views of Bichat, published toward the close of the last century, in his work entitled "Physiological Researches upon Life and Death." After quoting this distinguished physiologist's division of life into animal and organic, and his reasons for such division, he thus continues:—

"Now, although Bichat claimed for the organic life, that life common to both vegetables and animals, or rather for those parts or organs exercising the functions of the organic life, a 'mobility, or sensibility,' *i.e.* an irritability, and although he declared that upon the 'organic sensibility,' etc. depends the phenomena of digestion, circulation, secretion, exhalation, absorption, and nutrition, and that, moreover, this organic sensibility is common both to the plant and to the animal, and that 'the zoophyte enjoys it as perfectly as the most perfectly organized quadruped,' yet does it nowhere appear that he associated such organic life, or its specific qualities, as mobility, sensibility, or irritability, etc. with the ganglionic nerve structure."

Herein, then, is the peculiarity of the author's view; while, with Bichat, he regards irritability as a property common to both vegetable and animal tissues, upon which depends the strictly vital or organic functions, nutrition, secretion, etc., he does not receive it as an endowment of organic texture, but as a function of the ganglionic nervous system.

This view necessarily involves the admission that plants and cellular animals possess a nervous system analogous to the sympathetic. This fact, however, seems no difficulty to Dr. Davey. He says:—

"Our finite senses may never detect, even with the aid of art and science, what we shall be able to demonstrate as the analogue in vegetable substances of the ganglionic nervous system in man and the higher animals; but that such exists, though, perhaps, in a diffused and rudimental state, is, to my mind, a matter of demonstration—logical demonstration."

On this point we will only say the nerve cell and nerve fibril, the components of a nervous system, have specific characteristics by which we know them, and without which they cease to be such. A diffused nerve, therefore, is no nerve at all, and cannot be assumed as exercising the ordinary functions of a nerve. Moreover, the impossibility of detecting, with the highest microscopic powers, a structure which is usually seen with those of medium strength, is a warrantable evidence of its absence. We see reason, therefore, why we may reverse the author's "logical demonstration" and conclude that, because irritability is possessed by plants and cellular animals, in whom no nervous system can be discovered, that it is not solely a function of nerve structure. But let us leave this collateral question of the universal distribution of nerve tissue among organized beings, and consider upon what grounds the author regards irritability as a function of the sympathetic. We find his conclusions are chiefly drawn from the experiments of others, and mainly from those made by Le Gallois and Marshall Hall, to prove that the cerebrum and spinal marrow are independent organs; and those of Sir B. Brodie, instituted to show that muscular irritability is not derived from the spinal nerves. In order to appreciate the inferences of Dr. Davey, it is necessary to give an abbreviated history of these experiments. Le Gallois destroyed the spinal marrow in rabbits by thrusting a stylet through the whole vertebral canal; all life (reflex) ceased in the trunk; irritability alone remained. Dr. M. Hall experimented upon a decapitated frog, from which the viscera also had been removed. He says:—

"Now I beg here to repeat, the cerebrum, the centre of the spinal cord of nerves, and all the ganglionic, have been removed from this animal, and yet, when I pinch the extremity, it moves so as to be obviously perceptible at the remotest part of this theatre." "This influence passes not only from one extremity to the other, but it also passes from one set of extremities to the other set of extremities; thus it is quite plain that there is a nucleus of nervous matter between the two anterior extremities, and another nucleus between the two posterior extremities, by which these nervous links are united and associated in their motions one with another."

Upon these experiments Dr. Davey thus comments:—

"The irritability which Le Gallois tells us remained after the extinction of life in the rabbit, the spinal cord of which he destroyed, was doubtless due to the integrity of the ganglionic nervous system. This had been left intact, and was therefore in a position more or less, and in spite of the shock sustained by the system generally, to continue its specific functions; and hence the irritability of which the experimenter has spoken."

And again:—

"If the experiment on the frog, as above detailed by Dr. M. Hall, was of the value claimed for it, there would have occurred, on the extremities being pinched, not the mere movement 'so obviously perceptible to M. Hall,' but nothing more or less than the excito-motor phenomena in their stead; however, there were present only 'the motions without force,' that peculiar irritability which belongs to the organic nerve structure. The irritability observed by Le Gallois, in his experiment with the rabbit, and that manifested by the frog in the hands of Dr. M. Hall, was precisely of the same kind, although plainly much modified by the circumstances of the vivisection in either case."

This interpretation of the phenomena exhibited in these experiments seems so unwarranted by their history, as given above, that one is at a loss to surmise upon what grounds the author asserts their identity. Le Gallois clearly shows that reflex phenomena had ceased, the trunk lay motionless, but irritability, that motion without force, the product of direct stimulation applied to a part, remained; while Dr. Hall, implying that irritability was present, distinctly conveys that true excito-motor phenomena were also manifest. Stimulation of the surface produced movement of the limb, extending even to that of the opposite extremity. Does Dr. Davey imply a want of correctness in the statement of Dr. Hall as to the extent of movement? Surely the "frog experiment" has been too often repeated, with similar results, to justify such a conclusion; or, does he mean to suggest that reflex movement and irritability are the same? This, indeed, would seem to be his purpose, from the remarks which he makes on the following experiments of Sir B. Brodie:—

"First Experiment.—All tissues connecting the anterior extremity of a dog with its body were divided, except the axillary artery and veins, and twenty hours afterwards the muscles were found to contract strongly under the stimulus of the voltaic battery.

"Second Experiment.—The whole of the posterior part of the spinal marrow of a frog was removed, and five months afterwards the muscles of the hind-legs were found capable of powerful contractions."

The author says:—

"A comparison of these two experiments will show that the fore-leg of the dog and the hind-legs of the frog were, in all respects, alike; that is to say, their acquired anatomical conditions were on a par, and hence was it that, in both instances, that the 'contractile power,' the 'peculiar kind of sensibility,' *i.e.* irritability, or if Dr. M. Hall would prefer to have it so, the something which remained, was, on the application of a stimulus, so distinctly manifested in either case."

The "something which remained" here alluded to, is the reflex power which continued after the removal of the cerebrum and sympathetic ganglia, in the experiment of Dr. Hall, above cited. Now the essential apparatus of excito-motor phenomena is the "Diastaltic Nervous Arc," (Hall;) the impression must be conveyed from the surface to the spinal centre, and thence reflected to the muscles; in this consists the peculiarity of reflex action; but Dr. Davey cannot so regard it when he identifies the "something that remained," the function of the spinal centre, with muscular irritability, that contracts under stimulus, be it direct or reflected. The experiments above quoted, are those upon which the author relies to demonstrate the association of irritability with the sympathetic nervous system. Ignoring the possibility of its being an inherent property of tissue, he seems to have adopted a negative train of reasoning, considering it only necessary for his argument that he should prove that irritability is not a function of the cerebro-spinal nervous system. To this end even his illustrations do not seem most happily selected; for while two of them are conclusive against the spinal cord, the other two are almost as convincing against the supremacy of the sympathetic. To avoid the difficulty they suggest, Dr. Davey denies the assertion made by Dr. Hall, that the removal of the whole of the viscera of the frog is equivalent to the removal of the entire ganglionic system; and, on the other hand, affirms that this system is "intimately and indissolubly connected and incorporated with each fraction of one and all of the animal tissues," and, moreover, that it must never be forgotten that each ganglion belonging to the organic nervous system is held to be independent of every other; and, what is more, that each nerve proceeding from a single ganglion is, in a great measure, independent of that ganglion, and even that each point of such a nerve is independent of all the rest, and constitutes, *per se*, a distinct focus of nervous influence, an assumption sufficiently sweeping to enable the author to establish all he desires; but at the same time, in the absence of proof of such "fractional" relationship between the ganglionic nervous system and all tissues, a mere begging of the question. It is but little different, to declare irritability a direct property of tissue, or the property of an undemonstrable something "intimately and indissolubly connected and incorporated with it." While, therefore, we can, with Dr. Davey, "directly perceive that the experiments of Le Gallois, Hall, and Brodie, are just so many practical demonstrations of the persistence of a peculiar "irritability in the mutilated animal," we cannot see that they furnish any reasonable proof that it is "a specific function, or power, or quality exercised or possessed by the ganglia of the great sympathetic nerve."

Next in order, the author reviews the animal and organic functions, and takes up the consideration of the "formative power" as an effect or function of the ganglionic nervous system. After disposing of the opinions of Hunter, Abernethy, and Lawrence on the vital principle, and refuting the French doctrine that life is the result of organization, he thus proceeds:—

"Now both the organization and its functions may be said to be demonstrable to the senses, but the same cannot be said of the power to which organization is the instrument, though neither its presence nor its seat can be well doubted. Granting for the sake of argument, that the ganglionic nervous system is the source or origin of this power, or organizing principle, or creative force, it remains to show, the ultimate cause of this important part of the organism,—from whence did it receive its being?"

The ordinarily accepted doctrine that "life proceeds only from life, and that there exists no other but that which has been transmitted from one living body to another by an uninterrupted succession," is not received by the author, and although suggesting none other, he considers this view "clearly negatived" by the experiments of Messrs. Crosse and Weekes, on the production of acari in close atmospheres, under the influence of continued galvanic currents. Leaving this question of the origin of life with the acknowledgment that "it is not in the power of man to offer anything more than a very general reply," he returns to the consideration of the ganglionic nervous system as the habitation or organ of life. In support of this view he relies principally upon two points: its early formation in the embryo, and its integrity of development in certain monstrosities and idiots, in whom the organic functions had been very properly carried on, although the brain and spinal cord were deficient or entirely wanting. Admitting the anatomical correctness of these assertions, to wit, that the first solids of the embryo are portions of the ganglionic nervous system, and that the fully developed nerves in amyencephalous and brainless monsters belong exclusively to the sympathetic system, both of which have been denied, the conclusions of the author do not by any means follow; and while these interesting instances of arrested development undoubtedly prove that the "formation power," as manifested in the growth and maturity of the individual, may exist independent of the cerebro-spinal nervous system, the inference is not logical that, therefore, it must be an attribute or function of the sympathetic. On the other hand, the fact of those nerve-centres which ordinarily manifest such an influence upon its phenomena being proved not essential to its existence, would rather lead us to remove it altogether from the domain of nerve structure, and to this end the more recent researches of physiologists seem to tend, the disposition being to regard the vital properties of tissues as inherent in themselves, attributing to the nervous systems, cerebro-spinal and sympathetic, the control of the circulatory apparatus, by means of which the blood is distributed to each part of the economy according to its need, and thus indirectly the capability to increase or diminish the activity of the organic functions. Animal heat the author thinks essentially a vital process, and not a chemical one. The numerous theories advanced concerning its source, he deems unsatisfactory, but believes "the unprejudiced observer will feel satisfied with the views about to be submitted to him." He thus briefly states the opinions he does not entertain, and gives the view he regards with so much confidence :—

"If, then, the animal heat be not the product of any chemical change taking place, either in the lungs or in the secerning vessels—if it be not the result of the different capacities for caloric of the several fluid secretions of the body and their source, viz., the blood—if it do not depend on the moistening of the organic structures by their several fluids, nor on the influence of the cerebro-spinal system—from what source, I would ask, can the animal heat be derived, but from the ganglionic nervous system ?"

Without being forced to the conclusion by the cogency of Dr. Davey's reasoning, it does not seem improbable, as suggested by Dr. Carpenter, that the nervous system may, at times, act directly in producing animal heat, as it may do indirectly by accelerating the various chemical changes taking place in the body. In the electric eel and torpedo we see vital force manifesting itself in the form of electricity, and in their nerve-structure we find an arrangement for effecting rapid and extensive molecular changes: it is impossible not to associate these two facts in the relationship of function and organ. In the same way it is not improbable that heat may be generated by changes taking place in the nervous substance, and by the nerve-fibres distributed to all parts of the body. Be this as it may, however, there are facts which, on the other hand, clearly prove the nervous system is not the only source of organic heat; among these may be mentioned the increase of temperature after death, as observed by Dr. Dowler, and the development of heat in plants, during periods of active vitality. The last of the functions which Dr. Davey ascribes to the omnipotent sympathetic ganglia, is instinct. He looks upon it as nothing less than an animal function, varying with the construction of the nervous system exercising it, not an abstract quality, but one altogether dependent upon organization. One of the strongest facts upon which he bases his supposition that instinct is associated with the organic nervous system, is the occurrence of instinctive movements in monstrosities born without brain or spinal cord, and in some of the lower forms of animals in which only the ganglionic system is developed. The same idea apparently influences the author's conclusion here, as in consideration of the other functions, although it may be with more justice. He seems to have regarded all the phenomena of life as necessarily connected with nerve-structure, and finding the cerebro-spinal system absent, without a corresponding cessation of these phenomena, he concludes they must be functions of the sympathetic. The amyencephalous and brainless monsters of Drs. Hall and Lawrence have assisted largely, no doubt, in bringing the present volume to its publication. Thus ends the physiological chapter of the treatise, and we are enabled to understand why the author labored so strenuously in his preparatory anatomical remarks to establish priority of origin and influence to the sympathetic nervous system.

As an appendix to chapter ii., the author publishes a paper read by him before the British Medical Association, in 1854, entitled "On the Excito-motory or Diastaltic Nervous System of Dr. M. Hall." His views are sufficiently given in the following conclusions, which we find at the close of the article :—

"1. That the more complicated anatomy of the spinal cord suggested by Dr. M. Hall and sought for by Mr. Grainger, has not yet been realized.

"2. That the mere sensory and motor nerves or roots are themselves sufficient to the integrity of all those phenomena called excito-motory, which emanate from the spinal cord, or, in other words, that there is no necessity for any distinct division of the nervous (spinal) system, for the production of excito-motory phenomena.

"3. That the exercise of the functions of the spinal cord is due to the peculiar influence of the ganglionic nervous system exerted on its organism.

"4. That both the external and internal excito-motory phenomena are alike dependent on the vital stimulus imparted by the great sympathetic nerve; and in the former instance, through the instrumentality or medium of the medulla spinalis.

"5. That such is the relationship between the great nervous centres, viz., the brain, spinal cord, and the solar ganglion, that the second (viz., the spinal cord,) being at one time under the dominion of the first, *i.e.* the brain, and at another time under that of the third, *i.e.* the solar ganglia, and being thus intermediate, will now manifest a cerebral or voluntary power, (and this the result of consciousness, and, of course, volition,) and then (the antecedent circumstances being dissimilar) display one of an involuntary, automatic or instinctive, or excito-motory character; but this only of course when the organic nervous apparatus is in the ascendant and the brain comparatively quiescent; or, in other words, when sensation (spinal) and motion, and not consciousness and volition, (cerebral attributes,) are in operation, if I may be allowed so to express myself.

"6. That the great sympathetic nerve is endowed with a specific and independent power, to which both the brain and spinal cord, and with them, of course, their functions, are subordinate."

The chapter on Pathology is, of course, based upon the preceding views, and as the sympathetic was then regarded as the source of all vital operations, so it is now considered to be concerned directly or indirectly with every variety of abnormal action to which the organs of the body are subject. The author thinks pathologists have grossly overlooked the cause of disease in their zealous study of its effects; and although he would not ignore the investigation of morbid anatomy, he claims a more searching inquiry "into those changes in a portion of our organism (the ganglionic nerve-structure) which must have preceded and accompanied those more palpable and demonstrable to our senses." In the nosological arrangement of writers we are afforded merely a series of details of disease, while "we look in vain for any information concerning even the morbid state of the blood; and as for the organic nervous system—the moving power of the whole, the main-spring of the entire organism under circumstances of both health and disease—there is hardly any notice taken of it." In his aversion to these practices of others, Dr. Davey falls into the opposite extreme, and builds up his pathology without any reference to structural changes taking place in the sympathetic. He says:—

"It will not be doubted that if, as has been shown, the nutrition, secretion, exhalation, absorption, etc., as manifested throughout the whole organism and its parts, and as realized through the agency of the capillaries, are nothing more or less than the functions of the ganglionic system of nerves, it must follow that all deviations from the healthy state of the same functions can be accounted for in no other way than by referring them to certain changes in the *vital affinities* of the solids and fluids of the body, and which must be regarded as resulting from a depraved action of a part or the whole of the organic nervous system."

Among the many disorders attributed by the author to a diseased condition of the sympathetic, he thinks fevers afford the most satisfactory evidence, as in them all the vital functions are more or less interrupted or depraved. He looks upon the cold stage as closely allied to the collapse of cholera, and cites instances in his experience among the intermittents of Bengal and China, that were hardly less alarming. These examples of suspended vital activity manifest symptoms very like those produced by more unequivocal injuries to the sympathetic, as blows over the epigastrium, extensive bodily injuries, extreme fear, etc., and therefore should be referred to the same source. In proof that the syncope of collapse arising from these various causes is effected through the medium of the sympathetic, he cites the experiments of Marshall Hall, made for this purpose, when the influence of the brain and spinal cord was shut off by drugging with alcohol and laudanum, or by their removal from the body; and yet the crushing of a limb, or a blow upon the stomach, caused the circulation to cease instantly. Dr. Hall refers these effects to the ganglionic system. But besides fever and cholera, we have other diseases, a long and incongruous list, "apoplexia levis," hypochondriasis, atrophy of limited groups of muscles, chorea, angina pectoris, diabetes, dysentery, lepra, etc. etc. etc. through all of which the same idea of the author is apparent, that the first morbid impression is made upon the sympathetic, "inflammatory disorganizations" occurring afterwards "as the results of a subsequent abnormal action," the mere effects of the impression upon the sympathetic. We see no reason why Dr. Davey might not apply his hypothesis to all diseases as appropriately as to those he has enumerated; had he thus generalized, he would have condensed his chapter on Pathology to a paragraph, and in no way impaired its efficiency.

In the treatment of diseases affecting the ganglionic nervous system, the author advocates the use of free purgation, and of tonics and stimulants; the former to restore the suspended functions of the organic apparatus, the latter to support the vis vitæ. In cholera, this plan of practice first came to his notice under the direction of Mr. Twining, at the General Hospital, Calcutta, and since then he has not ceased to recognize it as the essential basis of all treatment. He says:—

> "Purgative medicines when given in cholera, so-called, *i.e.* when judiciously given, never act as purgatives, but they restrain the diarrhœa and effectually check the intestinal discharges; they act as restoratives to the normal character of the secretions, and so facilitate the formation of healthy fæcal matter."

In the selection of purgatives, Dr. Davey favors none as a specific, although the combination he used is composed of calomel, one grain and a half; compound extract of colocynth, five grains; oil of caraway, two minims. The pills were given every hour or two, according to the urgency of the symptoms, until the evacuations become yellowish or bilious in color, which happens generally after four or five doses. However, he cautions us against being too mindful of the drug and losing sight of the broad principle of purgation. In fever and the neuroses, the author advocates the free use of purgatives, thinking by this means to avoid many of these phenomena of exhaustion which result from the persistence of disease. Although willing to admit that Hamilton and Abernethy carried the purgative treatment of diseases, and of the neuroses especially, a little too far, he thinks the tendency of the day is to err upon the other extreme, to rely upon nature to the neglect of art; "that the present 'type of disease' does not require, nay, more, will not bear the same lowering treatment, viz., the bleeding, the purging, the puking, the starving which used to be pursued in certain cases, is most true; but to assert that the lancet is to be altogether proscribed and purgation fall into utter disuse, is to commit a capital offence against common sense and common experience." To support the vis vitæ, the author relies principally upon quinine in fever and cholera, and brandy and ammonia in the latter if the urgency of the case demands them. Twenty years ago, the author thinks the administration of quinine (bark) in fever was a more delicate question than now; that is, it was not so easy a matter to hit the precise time when this medicine would do good, and not aggravate the symptoms of the disease.

The "type of disease" has changed, so that now idiopathic febrile affections not only will bear the early administration of this drug, but the safety of patients actually demands it. The only antecedent measures necessary, are to rid the stomach and bowels of all offensive matters, and restore the tone of the excretory organs by more or less active purgation. In the subsequent parts of this chapter, the author adds much that is suggestive of the proper specific treatment of hypochondriasis, apoplexia levis, cardialgia, etc.; in fact, these few pages upon the treatment of disease contain most that is valuable in the work; they are practical and evidently the deductions of experience, and as such, are entirely independent of the hypothetical physiology to which they are attached. In conclusion, however, we may state that we have found the work, as a whole, very unsatisfactory; it fails entirely, we believe, to justify the author's views, and the fragments of truth incidentally scattered through its pages are wholly inadequate to compensate for the time and labor necessary for their discernment.

